# Invasive Serotype 35B Pneumococci Including an Expanding Serotype Switch Lineage

**DOI:** 10.3201/eid2402.170982

**Published:** 2018-02

**Authors:** Liset Olarte, Sheldon L. Kaplan, William J. Barson, José R. Romero, Philana Ling Lin, Tina Q. Tan, Jill A. Hoffman, John S. Bradley, Laurence B. Givner, Edward O. Mason, Kristina G. Hultén

**Affiliations:** Baylor College of Medicine, Houston, Texas, USA (L. Olarte, S.L. Kaplan, E.O. Mason, K.G. Hultén);; Ohio State University College of Medicine, Columbus, Ohio, USA (W.J. Barson);; University of Arkansas for Medical Sciences, Little Rock, Arkansas, USA (J.R. Romero);; Children’s Hospital of Pittsburgh of the University of Pittsburgh Medical Center, Pittsburgh, Pennsylvania, USA (P.L. Lin);; Northwestern University Feinberg School of Medicine, Chicago, Illinois, USA (T.Q. Tan);; University of Southern California School of Medicine, Los Angeles, California, USA (J.A. Hoffman);; Rady Children’s Hospital San Diego, San Diego, California, USA (J.S. Bradley);; Wake Forest School of Medicine, Winston-Salem, North Carolina, USA (L.B. Givner)

**Keywords:** pneumococci, pneumococcal disease, pneumococcal vaccine, serotype 35B, multidrug resistance, bacteria, antimicrobial resistance

**To the Editor:** We read with interest the article by Chochua et al. from the Centers for Disease Control and Prevention (*1*)*.* We appreciate that the authors cited our recent publication on the same topic from 8 children’s hospitals across the United States (*2*)*.* Our study encompassed pneumococcal serotype 35B invasive and noninvasive infections, spanning more than 2 decades. We believe, however, that their reference to our study needs some clarification. We did not report that clonal complex (CC) 156 was the major contributor to antimicrobial resistance among serotype 35B isolates, as stated by Chochua et al. We reported a predominance (69.2%) of 35B sequence type (ST) 558 among invasive isolates across the entire study period (before and after introduction of the 13-valent pneumococcal conjugate vaccine [PCV13]); 95% of the ST558 isolates were penicillin nonsusceptible. We noted that clonal expansion of ST558 was the major contributor to the increase in prevalence of serotype 35B, as did Chochua et al. Furthermore, we observed the emergence of 35B-CC156 after introduction of PCV13 and noted that 35B-CC156 isolates were multidrug-resistant (penicillin nonsusceptibility plus resistance to >2 non–β-lactam antimicrobial drugs), similar to previous observations of CC156 associated with other pneumococcal polysaccharide capsules (e.g., serotypes 9V and 14). Thus, the increase in multidrug resistance among serotype 35B isolates in the post-PCV13 era was strongly associated with the emergence of CC156.

Our conclusion that both clonal expansion and diversification had occurred in the post-PCV13 era is validated by the results of Chochua et al. During 2015–2016, we observed no further increase in serotype 35B. We agree with Chochua et al. that the emergence of serotype 35B is of concern and the development of a new generation pneumococcal vaccine is necessary. We will continue to monitor and report data regarding ongoing changes in pneumococci; although our study is not population based, we believe it provides reliable data that are useful for clinical, epidemiologic, and vaccine-related considerations.
